# New Insights Into the Circadian Rhythm and Its Related Diseases

**DOI:** 10.3389/fphys.2019.00682

**Published:** 2019-06-25

**Authors:** Yanling Xie, Qingming Tang, Guangjin Chen, Mengru Xie, Shaoling Yu, Jiajia Zhao, Lili Chen

**Affiliations:** Department of Stomatology, Union Hospital, Tongji Medical College, Huazhong University of Science and Technology, Wuhan, China

**Keywords:** circadian rhythm, synchronization, rhythm monitoring, influence factors, disorder

## Abstract

Circadian rhythms (CR) are a series of endogenous autonomous oscillators generated by the molecular circadian clock which acting on coordinating internal time with the external environment in a 24-h daily cycle. The circadian clock system is a major regulatory factor for nearly all physiological activities and its disorder has severe consequences on human health. CR disruption is a common issue in modern society, and researches about people with jet lag or shift works have revealed that CR disruption can cause cognitive impairment, psychiatric illness, metabolic syndrome, dysplasia, and cancer. In this review, we summarized the synchronizers and the synchronization methods used in experimental research, and introduced CR monitoring and detection methods. Moreover, we evaluated conventional CR databases, and analyzed experiments that characterized the underlying causes of CR disorder. Finally, we further discussed the latest developments in understanding of CR disruption, and how it may be relevant to health and disease. Briefly, this review aimed to synthesize previous studies to aid in future studies of CR and CR-related diseases.

## Introduction

**FIGURE 1:**
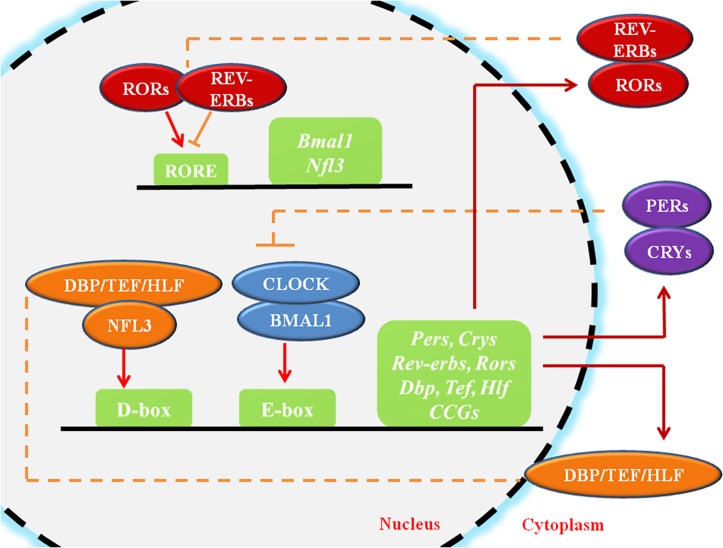
The molecular mechanism of circadian rhythms. CLOCK and BMAL1 activate the *cis*-acting element E-box to initiate the transcription of downstream genes such as Pers and Crys, while the accumulated PER and CRY proteins, in turn, bind to CLOCK/BMAL1 and switch them from an activated state to an inhibited state, suppressing the transcriptional activation of downstream genes. ROR/REV-ERB and DBP (TEF, HLF)/E4BP4, acting on other *cis*-acting elements such as RORE and D-box, participating in the regulation of the core feedback loop. CCGs refer to the clock-controlled genes. The circles represent proteins, the squares represent genes or clock-related elements, the red arrows represent transcriptional activation, and the orange lines with horizontal bars represent transcriptional inhibition.

Circadian rhythms (CR) are endogenous autonomous oscillators of physiological activities resulting 24-h day/night cycles, which allow organisms to adapt to a fluctuating environment (Reppert and Weaver, 2002; Dibner et al., 2010). The core pacemaker of CR lies in the SCN, which plays crucial roles in maintenance of systemic CR and regulates peripheral tissue clocks through secretion of endogenous regulatory factors (Dibner et al., 2010). The molecular clock of the CR system, which is present in all cells, is made up of oscillating clock-related proteins that compose TTFLs (Gekakis et al., 1998). The core TTFL is composed of the transcriptional activator proteins CLOCK and BMAL1, and the repressor proteins Period-1 (PER1), PER2, PER3, Cryptochrome-1 (CRY1) and CRY2 (Gekakis et al., 1998). Other loops are coupled to the core TTFL to maintain oscillation. The first sub-loop is composed of RORs and nuclear REV-ERB receptors. The second sub-loop comprises D-box-related genes, which include DBP, TEF, and HLF (Figure 1; Preitner et al., 2002; Sato et al., 2004). Moreover, recent studies have suggested that circadian regulation is heavily involved in gene expression. A considerable portion (approximately 10%) of genes expressed in cells or tissues have been found to display circadian oscillations, resulting in identification of these genes as “CCGs” (Duffield, 2003; Zhang et al., 2014).

**FIGURE 2:**
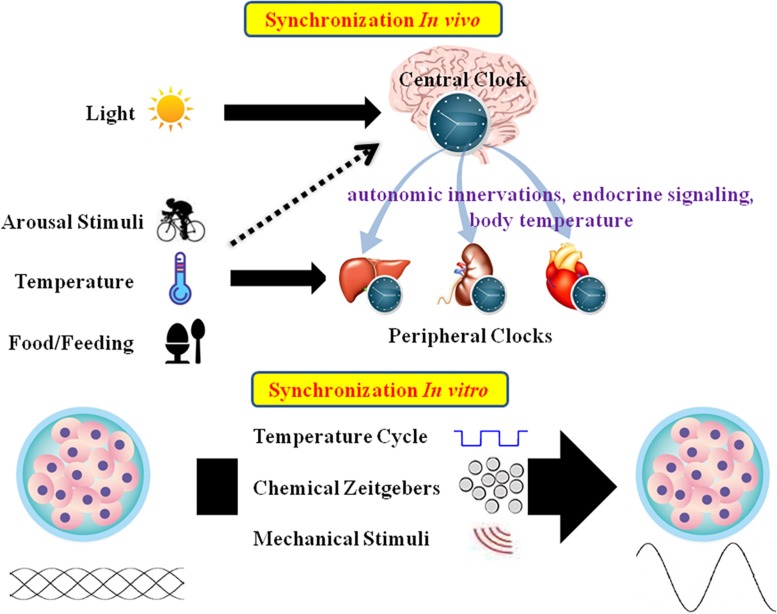
Schematic summary of *in vivo* and *in vitro* circadian synchronization. *In vivo*, the photic zeitgeber mainly entrains the central clock, which regulates the peripheral clocks through the internal timing cues including autonomic innervations, endocrine signaling and body temperature; the non-photic zeitgebers including arousal stimuli, temperature and food mainly entrain the peripheral clocks. *In vitro*, the circadian oscillations of cells or explants can be synchronized by temperature cycles, chemical factors (such as Dex, Fsk, or horse serum) and mechanical stimuli.

Mammalian tissues and cells have an autonomous circadian oscillator with a period of roughly 24 h. External stimuli are essential for maintaining the appropriate circadian oscillations (Bass and Takahashi, 2010; Roenneberg and Merrow, 2016; Tahara et al., 2017). *In vivo*, CR is mainly entrained by environmental signals such as light, food, and arousal stimuli. In the SCN, the circadian clock mainly responds to the LD cycle. In peripheral tissues, CR can be synchronized by food or temperature (Damiola et al., 2000; Hara et al., 2001; Buhr et al., 2010). Moreover, internal signals such as circulating hormones, cytokines, metabolites, sympathetic nervous activation, and body temperature are significant timing cues that regulate peripheral clocks (Albrecht, 2012; Menaker et al., 2013). *In vitro*, CR are difficult to observe due to lack of SCN signals. As such, external stimuli should be applied to induce CR in cultured cells or explants (Figure 2).

The field of chronobiology requires detection of features of CR in tissues or cells. Through chronological collection or luminescence monitoring, the basic data resulting from circadian fluctuations *in vivo* or *in vitro* can be accurately recorded. Approaches such as FFT (Moore et al., 2014), JTK-Cycle (Hughes et al., 2010), or autocorrelation (Levine et al., 2002) can be used to analyze the rhythmic features of CR. Subsequently, we introduced several circadian databases established in recent years.

Finally, we summarized *in vivo* and *in vitro* factors that can alter CR. *In vivo* factors include entrainments such as light, food, and temperature, while the *in vitro* factors include cell density (Noguchi et al., 2013), osmotic pressure, PH, mechanical stimulus, temperature, oxygen concentration, and microorganisms (Haspel et al., 2014). These factors can lead to circadian disruptions, and result in various diseases such as cancer, dysplasia, and metabolic and behavioral disorders (Takahashi et al., 2008).

## The Mammalian Circadian Clock System

The circadian clock system is the totality of all oscillators in organisms coupled to various physiological processes. This system generally consists of three parts in mammals including the input pathway, the core circadian clock, and the output pathway. The input pathway senses external timing signals, for example, light/dark, and sends information to the core circadian clock. The core circadian clock forms endogenous CR according to external time cues to allow for adaptation to the environment. Based on changes in the core circadian clock, the output pathway adjusts the physiological activities in various tissues and organs through neurohumoral regulation.

In mammals, the core pacemaker of the CR system exists in the SCN, which exhibits endogenous rhythmic oscillations both at the tissue and cell levels, plays a vital role in maintenance and alterations of CR, and provides outputs to peripheral tissues after synchronization by external time cues (Dibner et al., 2010). The SCN can be distinguished into two main areas: the VL core area and the DM shell area (Welsh et al., 2010). The core area mainly expresses the neuropeptide VIP, while the shell area expresses AVP. The VL-SCN, which controls essential physiological activities such as exercise, body temperature, heart rate, and hormone synthesis, serves to couple the circadian system. VIP is released periodically from the VL core region, binding to VPAC2 on the neuronal surface, resulting in cell depolarization and induction of PER1 and PER2 (Stepanyuk et al., 2014). VIP deficiency impairs synchronization of the cells, leading to weakening of whole-body rhythms (Loh et al., 2011). AVP-deficient rats exhibit weakened rhythms, but do not experience changes in circadian pacemaking. As such, the AVP-SCN is considered to function as the output (Mieda, 2019). In addition to VIP and AVP, other neurotransmitters such as glutamate and GABA, which are present in the SCN, conduce to regulate areas upstream and downstream of the circadian clock system (Morin et al., 2006). The SCN has historically been considered to be the only endogenous circadian clock with autonomous oscillations, and peripheral clocks were not believed to spontaneously oscillate, but could oscillate under pacing by the SCN. However, studies have demonstrated that the peripheral clocks also have the ability to oscillate autonomously and keep their internal rhythms (Balsalobre et al., 1998; Yoo et al., 2004). The predominant theory is that the SCN, as the master pacemaker, can drive peripheral clock rhythms (Dibner et al., 2010). Peripheral clocks are under the regulation of endogenous regulatory factors from the SCN. In an organized CR system, the connection between the SCN and peripheral tissues is a complex network, and the gene expression patterns of peripheral tissues are under the control of various complex factors including autonomic innervations, endocrine signaling, temperature, and local signals (Figure 2; Mohawk et al., 2012).

Within cells, the CR system regulates cell biological behaviors either directly through the TTFLs or indirectly (Figure 1). The core TTFL contains two transcription activators, CLOCK and BMAL1, which generate a heterodimer, bind to the *cis*-acting element E-box (5′-CACGTG-3′), and activates transcription of the *PER* and *CRY* genes at the beginning of the cycle (Gekakis et al., 1998). After several hours, PER and CRY proteins accumulate, dimerize, and generate a complex which translocates to the nucleus and inhibits the CLOCK/BMAL1 heterodimer (Xu et al., 2015). Other loops are coupled to the core TTFL to complete the oscillation. The first sub-loop comprises RORs and REV-ERB, which directly targets at CLOCK/BMAL1 or ROREs (Preitner et al., 2002; Sato et al., 2004). The second CLOCK/BMAL1-driven sub-loop contains the PAR-bZip factors DBP, TEF, and HLF. The repressor NFIL3 (or E4BP4), driven by the REV-ERB/ROR loop, interacts with these proteins at sites containing D-boxes (Mitsui et al., 2001; Gachon et al., 2004; Takahashi, 2017). These three interlocking TTFLs form 24-h cycles of transcription with diverse expression phases, resting with the combination of *cis*-elements (E-box, RORE, D-box) in the promoters and enhancers of specific CCGs.

## Circadian Rhythm Synchronizers

Nearly every eukaryote has a cell-autonomous circadian clock which exhibits 24-h physiological oscillations and can be influenced by external timing cues. These external timing cues, also called “synchronizers,” “zeitgeber,” or ”entraining agents” can reset the body’s circadian clock and place all cells at the same phase of circadian oscillation, a process called circadian rhythm synchronization. The word “ZT” is used to describe external cues that synchronize CR such as LD cycles or temperature cycles, and the word “CT” is utilized to describe timing without external signals (Li et al., 2017). In the field of chronobiology research, whether *in vivo* or *in vitro*, researchers use these entraining agents to synchronize CR of the experimental animals or cells. A variety of factors which act as synchronizers are summarized in Figure 2.

### Light

Most organisms acquire the time information through changing light intensity throughout the day to reset their own clock, referred to as “photic entrainment” (Roenneberg and Foster, 1997). In chronobiology experiments, light is often served as a stimulus to induce responses from the circadian clock. The light signaling cascades that entrain the circadian clock are fairly complex. *In vivo*, pRGCs receive light information, which is transmitted to the SCN directly through the RHT (Hattar et al., 2002; Ishida et al., 2005; Kalsbeek et al., 2006; van Diepen et al., 2015; Pilorz et al., 2016; Mouland et al., 2017; Astiz et al., 2019), resulting in regulation of the peripheral clocks by the SCN via secreting neurohumoral factors (Mohawk et al., 2012). The light-sensitive photopigment melanopsin (Provencio et al., 2000), is expressed in the pRGCs which mediate a series of responses to light (Hankins et al., 2008; Do and Yau, 2010; Schmidt et al., 2011). Stimulation of melanopsin causes activation of G protein coupled signaling cascades and the PLC pathway, leading to opening of TRPCs such as TRPC6 and TRPC7, Ca^2+^ influx, and cellular depolarization (Poletini et al., 2015). The input pathway to the SCN mainly depends on the monosynaptic RHT. The primary neurotransmitters in the RHT are glutamate, PACAP, SP, and aspartate (Ebling, 1996; Chen et al., 1999; Hannibal, 2002; Golombek et al., 2003; Hannibal and Fahrenkrug, 2004; Fahrenkrug, 2006). Glutamate, which binds to glutamatergic receptors such as NMDA or AMPA receptors, is the main signal for photic entrainment (Mikkelsen et al., 1993; Golombek and Ralph, 1996; Chambille, 1999; Peytevin et al., 2000; Michel and Colwell, 2001; Paul et al., 2005), and induces increased intracellular Ca^2+^ concentrations (Kim et al., 2005). PACAP, which activates PAC1 receptor, is also responsible for mediating synchronization to light (Hannibal et al., 1998, 2008; Shen et al., 2000; Colwell et al., 2004; Hashimoto et al., 2006). Ca^2+^ influx activates a range of kinases including PKA, MAPK, CaMK, PKCA, and PKG (Meijer and Schwartz, 2003). These kinases are involved in phosphorylation of CREB, which binds to cAMP response elements in promoters, resulting in transcription of clock genes such as *PER1* and *PER2* (Meijer and Schwartz, 2003). Moreover, transcription factors such as c-FOS (Travnickova-Bendova et al., 2002) and EGR1 (Riedel et al., 2018) also participate in regulation of the SCN clock through optical signals.

Based on RHT innervation and the neurochemical natures of the cells, the SCN is distinguished into two main subdivisions: the VL area (core area) and the DM area (shell area) (Moore et al., 2002). The VL-SCN, which contains VIP and gastrin-releasing peptide, is located above the optic chiasm, and receives most of its photic input from RHT innervation. In contrast, the DM-SCN region, where neurons contain AVP and calretinin, receives mostly neural signals from the hypothalamus, limbic areas, and the VL-SCN (Golombek and Rosenstein, 2010). VL-SCN neurons respond to photic stimuli during the subjective night and communicate with DM-SCN neurons through several neurotransmitters such as VIP, GRP, and SP (Best et al., 1999; Berson et al., 2002), resulting in synchronization of the DM-SCN to the expression of these proteins. Therefore, DM-SCN neurons depend on neuropeptide release from the VL-SCN, although they show stronger autonomous oscillations (Moga and Moore, 1997; Meijer and Schwartz, 2003; Antle and Silver, 2005).

### Arousal Stimuli

Other stimuli can synchronize CR, resulting in “non-photic entrainment.” Arousal stimuli are non-photic entrainments which include social interactions, exercise, restraint stress, and caffeine-induced arousal (Mistlberger and Skene, 2004). Different species respond differently to arousal stimuli. For example, during the daytime, wheel running or gentle handling cause a robust phase advance in locomotor activity rhythms of Syrian hamsters, whereas these stimuli do not arouse a similar phase advance in the behavioral rhythms of mice or rats (Antle and Mistlberger, 2000; Mistlberger et al., 2002). However, phase advance in peripheral clocks such as liver and kidney can be induced by restraint stress or running wheel exercise during the intermediate of the light phase in mice (Tahara et al., 2015; Sasaki et al., 2016). These studies suggested that under LD cycles, peripheral tissues directly respond to behavioral entrainment without influencing the SCN. However, in constant darkness during projected daytime, behavioral rhythms of mice can also be observed under several-hour daily wheel stimulation (Mistlberger, 1991). These results imply that the master clock in mice is primarily impacted by light rather than arousal stimuli.

A review by Golombek and Rosenstein (2010) showed that arousal stimuli transmit to the SCN through two major pathways. The GHT from the thalamic IGL, employs NPY, GABA, and endorphins as neurotransmitters, and plays a role in activation of the Y2 receptor/PKC pathway. The other is the serotonergic median raphe nucleus projection to the SCN, where serotonergic effects are mediated by 5-HT_1A/7_ receptors and PKA activation (Golombek and Rosenstein, 2010). The mechanisms responsible for behavioral entrainment of peripheral clocks include physiological factors such as glucocorticoids, sympathetic nerves, oxidative stress, hypoxia, PH, cytokines, and temperature (Tahara and Shibata, 2018).

### Food/Feeding

The CR of mammals can be synchronized by light, and thus the central clock for light response, the SCN, is considered a light-entrainable oscillator. Food is a non-photic stimulus that can reset the circadian rhythm. Many studies have confirmed the existence of a FEO, but the anatomical location(s) and molecular timekeeping mechanisms of the FEO have not been determined. In the case of mammalian research, food is provided and consumed within a few hours. This condition is called time-restricted feeding, or temporal food restriction, and is frequently used to study FAA (Challet et al., 1998). FAA refers to the output of the FEO, which appears under time-restricted feeding, but disappears under *ad libitum* access, and reoccurs during the following fasting (Pendergast and Yamazaki, 2018). Mice allowed access to a standard diet *ad libitum* typically ingest 60–80% of their daily food intake at night.

Feeding-fasting signals reset the circadian clocks in the peripheral tissues by causing periodic availability of many circulating macronutrients (Woods, 2005). For example, ingestion causes release of insulin into the blood and induces clock gene expression in insulin-sensitive tissues (such as liver, adipose, and muscle) (Oike et al., 2014). Feeding also enhances blood glucose levels, and high glucose concentrations can down-regulate the expression of *PER1* and *PER2* in fibroblasts (Hirota et al., 2010), and indirectly regulate AMPK, which controls the stability of CRYs (Lamia et al., 2009). Recent evidence showed that the liver may be a communication center of the FAA synchronization signal. Under time-restricted feeding, the liver increased βOHB production through regulation of *Cpt1a* and *Hmgcs2* by *Per2*, which was then used as a signal to cause animals to anticipate feeding time (Chavan et al., 2016). In addition, food restriction may alter the anabolic/catabolic cycles of tissues, which may affect the cellular redox state (Rutter et al., 2002) and further influence the circadian feedback loop (Mendoza, 2007). For instance, NADH and NADPH, classical cofactors for intracellular redox reactions, can promote binding of CLOCK/BMAL1 and NPAS2/BMAL1 dimers to DNA, while their oxidized forms, NAD(P)+, inhibit this binding (Rutter et al., 2001, 2002; Schibler and Naef, 2005). Furthermore, restricted feeding may influence body temperature cycles, which have been confirmed to entrain peripheral clocks (Brown et al., 2002).

Although the central oscillators are not affected by temporal food restriction during the light phase of the day (Damiola et al., 2000; Stokkan et al., 2001), SCN-derived physiological rhythms such as locomotor activity and body temperature can be entrained by caloric restriction (also called hypocaloric diet, characterized by caloric intake reduction to 60% of the animal’s normal daily food intake) under LD cycles (Challet et al., 1997) or time-restricted feeding in constant darkness (Holmes and Mistlberger, 2000). Although the mechanisms by which caloric restriction affects the SCN clock has not been characterized, previous studies suggested that receptors of metabolism-related hormones such as insulin, leptin, and ghrelin, which exist in SCN cells, may involve in synchronizing the SCN (Guan et al., 1997; Zigman et al., 2006). Moreover, feeding regulates brain structures that project straightly to the SCN such as the DM hypothalamus and the arcuate nucleus through orexin and ghrelin (Akiyama et al., 2004; Mieda et al., 2004; LeSauter et al., 2009; Moriya et al., 2009; Acosta-Galvan et al., 2011). Moreover, glucose influences the neural activity phase *in vitro* SCN slices (Hall et al., 1997). Further researches are required to evaluate the involvement of these factors and their probable impacts on the SCN.

### Temperature

Temperature is a non-photic synchronizer with a weaker synchronization effect than that of light. Roberto Refinetti found that only 60–80% mice can be synchronized by ambient temperature cycles, and stable entrainment takes longer in response to temperature cycles than LD cycles (Refinetti, 2010). Though multiple physiological processes rely on ambient temperature, the CR system has a significant feature called temperature compensation, in which circadian oscillations remain resistant to temperature changes, resulting in the period length still maintaining approximately 24 h despite ambient temperature changes (Isojima et al., 2009; Narasimamurthy and Virshup, 2017). In addition, the SCN clock does not respond to temperature stimuli, whereas cells and tissues outside of the SCN can be synchronized by temperature fluctuations (Brown et al., 2002; Buhr et al., 2010; Ohnishi et al., 2014). The reason why the SCN is resistant to temperature changes could be that the circadian clocks in the SCN cells have robust couplings. After uncoupling of SCN cells using tetrodotoxin or nimodipine, temperature sensitivity was detected (Buhr et al., 2010). Because temperature is a weak synchronizer, it is not typically used as a zeitgeber in animal experiments.

The phase shift of the clock in response to simulated body temperature fluctuations has been demonstrated *in vitro* (Brown et al., 2002; Buhr et al., 2010; Saini et al., 2012; Dudek et al., 2017), and the amplitude of circadian gene expression was enhanced by temperature cycles (Dibner et al., 2009; Sporl et al., 2011; Dudek et al., 2017). Mammalian cells sense temperature fluctuations through a series of temperature-stimulated TRP channel subfamily members called thermo-TRPs, each of which is activated in a narrow thermal range (Poletini et al., 2015). The intrinsic mechanism of temperature effects on CR may be mediated by HSF1 and CIRBPs (Ki et al., 2015). HSF1 is a circadian transcription factor which binds the heat shock element sequence, causing circadian activation of downstream promoters such as Per2 (Kornmann et al., 2007; Reinke et al., 2008; Tamaru et al., 2011). CIRBP-mediated post-transcriptional regulation allows high-amplitude clock genes express, including that of the core clock gene *CLOCK* (Ki et al., 2015).

### Chemical Factors

*In vitro* experiments lack the humoral and neuronal factors that can act as entrainments *in vivo*. As such, cultured cells or explants are desynchronized and circadian oscillations are absent. Thus, to observe endogenous oscillations of cells or explants *in vitro*, chemical factors with similar effects to *in vivo* entrainments are required.

Many chemical factors can function as synchronizers. It has been reported that glucocorticoid, a kind of anti-inflammatory hormones released by the adrenal cortex, serves as a vigorous synchronizer of peripheral tissues (Balsalobre et al., 2000a, b; Yamamoto et al., 2005; Segall et al., 2006; So et al., 2009; Cheon et al., 2013). Dex, an artificial glucocorticoid, exposure for 1 h can be used to restart the oscillations of circadian clock genes (Balsalobre et al., 2000b; Wu et al., 2008). Glucocorticoids activate GRs, which bind to the GREs on the promoters of core clock genes such as Per1, Per2, and E4bp4, thereby directly activating the core clock cycle, or by activating the transcription factor HNF4alpha, which targets downstream rhythmic genes without GRE elements (Reddy et al., 2007; Cheon et al., 2013). Dex cannot be used for SCN synchronization, because the SCN does not express GRs.

A study performed in 1998 indicated that serum shock could induce the rhythmic expression of *Perl*, *Per2*, *Reverb-*α, *Dbp*, and *Tef* in cultured rat fibroblasts (Balsalobre et al., 1998). Thereafter, serum shock was used to synchronize the circadian oscillations of various tissues, immortalized cells, and fibroblast cells. This study suggested that various factors in the blood could stimulate rhythmic oscillations. Previous reports showed that various factors such as such as EGF (Izumo et al., 2006), calcium (Balsalobre et al., 2000b), glucose (Hirota et al., 2002), PGE2 (Tsuchiya et al., 2005), 1α,25-dihydroxyvitamin D3 (Gutierrez-Monreal et al., 2014), and atomoxetine (O’Keeffe et al., 2012) can synchronize clock genes. Moreover, serum shock can induce Ser/Thr phosphorylation of CLOCK through the Ca^2+^-dependent PKC pathway (Shim et al., 2007), or activate the p42/44 MAPK pathway in a manner similar to that observed in response to light pulses (Ginty et al., 1993; Yagita and Okamura, 2000), which may cause resetting of the clock. In addition, a previous report showed that blood-borne signals activate GTPase RhoA, which promotes G-actin polymerization into F-actin, resulting in release of MRTFs into the nucleus, thus regulating the transcription of clock genes (Esnault et al., 2014).

Another common chemical synchronizing agent, Fsk, has a similar mechanism to that of serum. Fsk directly activates AC, which activates CREB through phosphorylation by promoting the synthesis of cAMP and activating PKA indirectly (Yagita and Okamura, 2000).

### Mechanical Stimuli and Oxidative/ Hypoxia Stress

A recent study showed that intermittent uniaxial stretching of bone marrow-derived mesenchymal stem cells, DPSCs, and adipose tissue-derived mesenchymal stem cells can reset their CR, resulting in a synchronization effect similar to that observed in response to Dex treatment (Rogers et al., 2017). Changing media also could reset cellular CR (Yeom et al., 2010; Guenthner et al., 2014). Mechanical stimuli provide researchers with alternate mechanisms to reset the circadian clocks of cells, such as DPSCs, which cannot be synchronized by other methods. However, the mechanisms by which mechanical stimuli induced synchronization are still unclear. It might be involved the RhoA pathway, by which short-duration fluid shear force can induce changes expression levels of clock genes in osteoblasts (Hamamura et al., 2012).

Oxidative or hypoxic stimuli may lead to circadian clock entrainment. *In vitro*, oxidative stimulation via hydrogen peroxide synchronizes cellular circadian oscillations in dose- and time-dependent manners (Tamaru et al., 2013). *In vivo*, phase shifts in peripheral clocks such as those in the kidney and liver are observed after hydrogen peroxide injection (Geerdink et al., 2016). However, whether the master clock responds to oxidative stress is unknown. Oxygen cycles (12-h 5%:12-h 8%) synchronize cellular clocks via a key transcription factor in cultured fibroblasts, HIF1α, which has similar a similar PAS domain to CLOCK and BMAL1 (Adamovich et al., 2017). Furthermore, at the onset of a 6-h-shifted dark period, hypoxic stimulation (14% O_2_) for 2 h advanced phases of locomotor activities in response to a new LD cycle (Adamovich et al., 2017). These results suggested that both central and peripheral oscillations can be reset by hypoxic stimulation. Taken together, oxygen signals may induce circadian synchronization *in vitro*.

## Common Synchronization Methods Used in Lab

### Synchronization *in vivo*

#### Light Stimuli

Most researchers choose light to synchronize CR in animals. Most studies utilize a rectangular LD cycle, which includes sudden transitions from light/dark to dark/light. Under laboratory condition, the 24-h LD cycle can be represented in various ways, such as a short photoperiod (shorter than 12-h light per day, e.g., 6L:18D), an equinox photoperiod (12-h light per day, i.e., 12L:12D), and a long photoperiod (longer than 12-h light per day, e.g., 18L: 6D). In nature, however, the transition between light and dark is not abrupt, and is characterized by gradual changes in light intensity and spectral composition. Mimicking natural conditions as closely as possible requires a light transition time (usually 0–2 h) in which the log of light intensity increases (or decreased) over time from 0 to 100 lux, and these changes can be implemented at any point during the transition from light to dark or dark to light. Different photoperiods lead to different waveforms of the SCN population rhythm. In SCN, short photoperiods result in waveforms that are slim and high-amplitude, while long photoperiods lead to the wide and low-amplitude waveforms (Inagaki et al., 2007; VanderLeest et al., 2007). Increasing the photoperiod compresses the activity phase of mice, but this effect is weakened by a long twilight duration (2-h) (Comas and Hut, 2009). In addition, light intensities impact the phase angle of entrainment and the velocity of re-synchronization to a new LD cycle. Specifically, low light intensity results in a longer phase angle (Wright et al., 2005), decreasing the rate of re-entrainment. If light intensity is insufficient, entrainment may fail (Leloup and Goldbeter, 2013).

An equinox photoperiod is used to study normal CR in animals. However, sometimes the 16L:8D cycle is used for mice because the locomotor period of mice is usually longer than 12 h (Albrecht and Foster, 2002). Furthermore, to observe the autonomous rhythms of animals, constant darkness (i.e., dark-dark, or DD condition) after a stable entrainment of 7–10 days in the LD cycle is required to avoid the effects of light (Albrecht and Foster, 2002). However, genetically engineered mice with no functional clock such as *Per1/Per2* and the *Cry1/Cry2* double-mutant mice (van der Horst et al., 1999; Zheng et al., 2001) seem to lose circadian rhythmicity in DD conditions, but show obvious rhythmic activity under LD conditions. The condition in which rhythmicity is induced by the LD cycle but not by the endogenous clock is called masking (Mrosovsky et al., 2001).

Longer light durations are used to affect the frequency of rhythms, whereas short light pulses are used to induce phasic entrainment of the clock (Daan, 1977; Roenneberg et al., 2003). The durations of short light pulse experiments are usually 15–30 min. Light pulses during the activity period of the mouse (subjective night) induce phase shift (advance or delay) of the clock, while light stimulus during the resting period (subjective day) alters the phase of the circadian clock (Johnson, 1999; Johnson et al., 2003). Use of this phenomenon allows for production of a phase response curve. In fact, using one or two light pulses to determine the frontiers of the subjective day and night of animals (also called “skeleton photoperiod”) can reduce negative masking (Lewy et al., 2001; Kennaway, 2004).

To study biological behaviors resulting from disrupted CR, jet lag protocols that advance the LD cycle (delaying may mask the onset of activity) can be used (Cao et al., 2013; Jagannath et al., 2013; Yamaguchi et al., 2013). In addition, constant light conditions can prolong the clock period compared to constant dark conditions, leading to a daily phase delay of activity in a light intensity-dependent manner. The sensitivity of these responses can be expressed as an IRC (Daan and Pittendrigh, 1976). Different wavelengths of light have different effects on phase, resulting in multiple IRCs that enable formation of an action spectrum (Peirson et al., 2005; Foster et al., 2007).

#### Time-Restricted Feeding

In addition to LD cycles, time-restricted feeding is used to examine the function of food intake timing on the circadian clock, and to synchronize peripheral oscillators, particularly in studies of metabolism-related tissues such as the liver, adipose, pancreas, and kidney. In these experiments, food access timing is restricted to between 2 and 12 h during the day or night. Within a few days, the rodents begin to predict the timing of food arrival and eat their meals during the period of food supply. If the period of food supply is less than 6 h, the animals cannot eat an equivalent amount of food compared to those allowed food *ad libitum*. When the period of food supply is over 8 h, the animals ingest nearly an equal amount of calories as those allowed food *ad libitum*. Thus, time-restricted feeding that allows greater than 8 h of access to food is an effective method to examine the role of food timing entrainment in response to changes in nutrition quality or quantity (Manoogian and Panda, 2017).

Because the synchronization effects in response to temperature and arousal stimuli are comparatively weaker than those in response to light, these factors are not commonly used *in vivo* experiments (unless the purpose is to study the synchronization effects of these two factors), but researchers should work to control for these factors to prevent interference with the experimental results.

### Synchronization *in vitro*

In cultured cells or explants, chemical synchronization reagents such as Dex, Fsk, or 50% horse serum are commonly used to reset circadian oscillations. As previously discussed, Dex is unable to synchronize SCN cell lines or explants due to lack of GRs. Different synchronization reagents have different abilities to induce clock gene oscillations. Mariko Izumo et al. compared the synchronization effects of ten different compounds in Rat-1 fibroblasts, and found that horse serum, Dex, Fsk, and EGF induced the largest oscillatory amplitudes, among which Fsk and Dex induced the highest-amplitude rhythms (Izumo et al., 2006). In addition, Dex-induced rhythms reached the strongest amplitude 30 min after administration, faster than Fsk, and the amplitude of Dex-treated cells was consistently higher than that of Fsk (Izumo et al., 2006; Takarada et al., 2017). These discrepancies may have been by reason of differences in synchronization mechanisms (Izumo et al., 2006). In addition to these chemical synchronization approaches, other synchronization approaches should be considered under some circumstances. For instance, when synchronization reagents are not readily permeable (e.g., some dense explants or cells are grown in 3D materials), temperature cycles could be a good alternative. An example protocol is as follows. Cells or explants are cultured for 2–3 days with a square-wave temperature cycle of 12-h low temperature (35.5–36.5°C) and 12-h high temperature (38.5°C). After 2–3 days, when cells return to a constant temperature condition of 37°C (CT0), the rhythm of synchronized cells or explants can be monitored (Dibner et al., 2009; Sporl et al., 2011; Dudek et al., 2017; Yang et al., 2017; Williams et al., 2018). Similar to Dex, temperature cycles cannot be used to synchronize SCN oscillations because of robust couplings in the SCN. Newly discovered mechanical stimulation synchronization may also be useful, but specific instances where this is the case have not been identified.

Some other “synchronization” methods such as centrifugal washing and serum starvation (Banfalvi, 2017) are used to synchronize the cell cycle so that all cells are in the same phase of the cell cycle. The cell cycle is defined as the whole process from the end of last round of mitosis to the beginning of next round of mitosis, which contains four distinct phases: G1 phase (RNA and ribosomes synthesis), S phase (chromosome replication), G2 phase, and M phase (mitosis). G0 phase refers to the quiescent state in which cells have temporarily stopped dividing. Similar to the CR system, the cell cycle appears to be an oscillator, where the periodic expression of cyclins controls cell cycle progression in a sequential and unidirectional manner. Both the cell cycle and the CR system exhibit sequential phases in the transcription/translation of genes and modification/degradation of proteins. As two basic periodic processes that occur within a day, these two systems are interrelated and interact. Control of the cell cycle progression by the circadian clock is known as circadian gating of the cell cycle. Previous reports have indicated that the CR system regulates cell cycle progress both at the G1/S (Geyfman et al., 2012; Kowalska et al., 2013) and at G2/M (Matsuo et al., 2003; Tulina et al., 2014) transitions. In contrast, the cell cycle can regulate the circadian clock as well. Nagoshi et al. (2004) indicated that cell division could influence the circadian clock by impacting the concentrations of PER/CRY protein complex. Bieler et al. (2014) showed that the cell cycle oscillator has a significant effect on the clock oscillator, resulting in a robust synchronization of both cycles in NIH3T3 cells.

Studies have indicated that the circadian oscillator and the cell cycle oscillator are bidirectionally coupled. For example, the cell-cycle duration is usually within the range of the circadian period in many mammalian cell lines (Glass, 2001). When cells are arrested to the same stage of the cell cycle by methods such as serum starvation (retaining cells at the G0/G1 phase), we observed some synchronization of CR (Nagy et al., 2016). Moreover, circadian synchronization induced by Dex leads to clusters of dividing cells, suggesting that the cell cycle is synchronized through the circadian clock (Feillet et al., 2015). Coupling (also called phase locking or mode locking) is defined by the coupling ratio p:q, in which p cell cycles finish during q circadian cycles (Glass, 2001). In the loss of external timing signals, the circadian clock and cell cycle display phase locking with a ratio of 1:1 (Bieler et al., 2014). However, other studies have shown that CR are not exactly synchronized with the cell cycle synchronization, and while there may be coupling between CR and the cell cycle, the coupling ratio is not always 1:1, and can be altered with increases in serum concentration or cell differentiation (Feillet et al., 2014; Matsu-Ura et al., 2016). In conclusion, researchers can choose different synchronization methods based on the advantages and disadvantages of various synchronizers to address different experimental questions (summarized in Table 1).

**TABLE 1:** Summary of the effects and mechanisms *in vivo* and *in vitro* zeitgebers.

**Zeitgebers**		**Effect and Application**	**Mechanism**	**References**
*In vivo*	Light	The strongest zeitgeber; Mainly entrain to SCN; 12L:12D to study normal rhythm	pRGCs, RHT; Glutamate and NMDA or AMPA receptors; PACAP and PAC1 receptors; Ca^2+^-dependent PKC and CREB pathway, MAPK pathway, c-FOS, EGR1	Mikkelsen et al., 1993; Golombek and Ralph, 1996; Hannibal et al., 1998, 2008; Chambille, 1999; Peytevin et al., 2000; Shen et al., 2000; Michel and Colwell, 2001; Hattar et al., 2002; Travnickova-Bendova et al., 2002; Colwell et al., 2004; Ishida et al., 2005; Paul et al., 2005; Hashimoto et al., 2006; Kalsbeek et al., 2006; Poletini et al., 2015; van Diepen et al., 2015; Pilorz et al., 2016; Mouland et al., 2017; Riedel et al., 2018; Astiz et al.,2019
	Arousal Stimuli	Weaker than light	GHT from IGL (NPY, GABA, endorphins; Y2 receptor/PKC pathway); serotonin (5-HT1A/7 receptors and PKA activation)	Golombek and Rosenstein,2010
	Food/Feeding	Mainly entrain to peripheral tissues; Restricted feeding to study metabolism-related rhythm; Weaker than light	Causing periodic availability of many circulating macronutrients such as insulin and glucose; Redox state changes (NADH and NADPH); Body temperature cycles	Rutter et al., 2001, 2002; Schibler and Naef, 2005; Woods, 2005; Lamia et al., 2009; Hirota et al., 2010; Oike et al.,2014
	Temperature	Weaker than light	Thermo-TRPs, HSF1 and CIRBPs	Ki et al., 2015; Poletini et al.,2015
*In vitro*	Temperature cycles	Long synchronization time (2–3 days); Used when the chemical synchronizer is impermeable; Do not work on SCN	Thermo-TRPs, HSF1 and CIRBPs	Ki et al., 2015; Poletini et al.,2015
	Serum Shock	Short synchronization time (0.5–2 h); The effect is not as good as Fsk and Dex	Ca^2+^-dependent PKC and CREB pathway, MAPK pathway, Rho-actin signaling and MRTF	Ginty et al., 1993; Yagita and Okamura, 2000; Shim et al., 2007; Esnault et al.,2014
	Fsk	Short synchronization time (0.5–2 h); The effect is between serum and Dex	cAMP/PAK-CREB pathway	Yagita and Okamura, 2000; Izumo et al.,2006
	Dex	Short synchronization time (0.5–2 h); Best effect in comparison to Fsk and serum in, peripheral tissues; Do not work on SCN	Glucocorticoid receptors/ HNF4alpha - GREs	Balsalobre et al., 2000b; Reddy et al., 2007; Wu et al., 2008; Cheon et al.,2013
	Mechanical Stimuli	Long synchronization time (2–3 days); An alternative when other synchronizers do not work		Rogers et al.,2017
	Oxidative/Hypoxia Stress		HIF1α (Hypoxia Stress)	Geerdink et al., 2016; Adamovich et al.,2017

## Circadian Rhythm Monitoring and Detecting Methods

### Rhythm Monitoring Methods

Since the discovery of CR, researchers have used various methods to monitor this phenomenon. In animal studies, a wheel-running system with passive infrared sensing elements is utilized to monitor the rhythm of locomotor activity (Albrecht and Foster, 2002). RNA or proteins are extracted from cells or tissues by successive sampling to determine the oscillatory patters of clock genes *in vitro* or *in vivo*. This chronological collection approach is severely limited by many practical issues (Gaspar and Brown, 2015). After sampling chronologically, detection of gene fluctuations at the transcriptional or translational levels requires complex steps including extraction, RNA reverse transcription/protein denaturation and PCR/immunoblotting, which may increase the error of the results. In addition, since detection is not in real-time, time intervals are between sample collections at different time points may result in maximum or minimum values not being captured. Moreover, chronological collection is relatively labor-intensive, making it difficult to decreasing errors by enhancing temporal resolution (Sellix et al., 2010). Furthermore, it is difficult to distinguish the defects whether come from intercellular synchrony or cell-autonomous clock function because the individual cellular clocks cannot be monitored (Sellix et al., 2010).

In recent years, there has been rapid progress in bioluminescence reporter technologies and specialized dynamic imaging techniques, allowing for real-time quantitative monitoring of rhythmic oscillations, resulting in advances in the field of chronobiology (Ballesta et al., 2017). Since the early 1990s, luciferases have been utilized as indicators of circadian clock genes (Welsh and Kay, 2005). By fusing the promoter of a target gene to the firefly luciferase reporter gene at the cellular or organismal level, stable reporter cell populations or stable reporter transgenic mice can be generated (Yamazaki et al., 2000; Izumo et al., 2003). *In vitro*, after synchronizing the reporter cells or tissue explants, the samples are placed in medium containing luciferin (the substrate for luciferase). Apparatuses with PMTs are used to detect oxidation of luciferin by luciferase (Yoo et al., 2004), allowing for real-time monitoring of gene expression. By inserting an optical fiber into the SCN of a PER1-luciferase transgenic mouse, the *in vivo* central clock oscillations can be recorded in real-time (Yamaguchi et al., 2001). In addition, by subcutaneously injecting luciferin into Per2::luc mice (Tahara et al., 2012), or by injecting Adv-Bmal1-luc viral vectors targeting hepatocytes via tail vein injection in combination with intraperitoneal injection of a fluorescein solution (Saini et al., 2013), oscillations of peripheral clocks such as the liver, kidney, and submandibular glands can be surveilled *in vivo*. Apparatuses such as fluorescent imaging systems, cooled CCD cameras, or RT-Biolumicorders (designed by Saini et al., 2013) are used to monitor circadian oscillations. The CCD camera, which can detect a single photon, allows for single-cell fluorescence rhythm monitoring *in vitro* or *in vivo* (Welsh and Kay, 2005; Sellix et al., 2010; Lande-Diner et al., 2015).

The luciferase reporter system can be employed to supervise circadian oscillations at the transcriptional level in real time. In these experiments, the generated luminescence is very weak and the background luminescence is nearly zero, which has the advantage of preventing phototoxicity related to long-term illumination (Sellix et al., 2010). However, the luciferase reporter system is not suitable for measuring the fluctuations of clock proteins and their sub-cellular localization, and their requirement for luciferin as substrate can result in luciferase chemistry-dependent artifacts (Smyllie et al., 2016). Smyllie et al. (2016) generated a PER2::VENUS mouse, which expresses the fluorescent fusion protein PER2::VENUS, allowing for dynamic acquisition of information regarding PER2 in individual mammalian cells. The PER2::VENUS half-life is similar to that of PER2::LUC, but it directly measures the circadian protein PER2 instead of indirectly measuring enzymatic luciferase activity. As such this strategy may provide a more accurate estimate of PER2 stability and rhythm (Smyllie et al., 2016).

Taken together, a number of methods for monitoring CR are available and can be chosen according to experimental conditions and the limitations and benefits of each of the above introduced monitoring methods (summary in Table 2).

**TABLE 2:** Summary of circadian rhythm monitoring methods.

**Monitoring method**	**Equipment**	**Advantage**	**Disadvantage**
Monitor the rhythm of locomotor activity	Wheel-running system with passive infrared sensing elements	Monitor locomotor activity in real time	Need specific equipment;
Chronological collection	RNA-sequencing, Microarray, qRT-PCR, Western blot	Dot not need specific equipment	Complex steps and time interval detection increase the errors; Labor-intensive; Single-cell detection is difficult to achieve
The luciferase reporter system with real-time bioluminescence monitoring	Apparatuses with PMTs, CCD camera	Real-time detection; Not labor-intensive; Can detect single cell	Need specific equipment; Can not measure the fluctuations of clock proteins and their sub-cellular localization
Fluorescent protein fused with clock protein and real-time bioluminescence monitoring	Apparatuses with PMTs, CCD camera	Real-time detection; Not labor-intensive; Can detect single cell; Can measure the fluctuations of clock proteins and their sub-cellular localization	Need specific equipment;

### Rhythmicity Detecting Methods

One of the basic problems in CR research is detection and analysis of the rhythmicity of measured data. Following collection of physiological data from a cell over many days, we are able to evaluate periodicity. Rhythmic data has several basic characteristics. Period is defined as how often the cycle is repeated. If the period is 24 h, the data pattern of oscillation reoccurs every 24 h. Phase refers to the time point of peak or trough expression, which reflects the timing in the individual tissues or cells. Phase angle of entrainment means the appropriate phase relationship between the endogenous CT and external environmental time. Amplitude is the magnitude of oscillation between peak and trough. If the data is rhythmic, we must evaluate more parameters than whether the data vary over time. We should also estimate whether the waveform meets the characteristics of a periodic function, such as a cosine function, as determined by its amplitude, period, and phase (Herzog et al., 2015). FFT is the most common method for detection of periodicity, and generally assumes that the fundamental rhythm is in the form of several cosine waves. This method fits the data to cosine functions with different periods, amplitudes, and phases (Moore et al., 2014). The periodicity in the data is fitted to the highest-amplitude cosine function (Herzog et al., 2015). Another method is JTK-Cycle, which is a non-parametric statistical algorithm for identifying and characterizing rhythmic expression in large datasets. It can reliably detect the transcripts in datasets with oscillating abundance because of its statistical power, specificity, accuracy, and precision (Hughes et al., 2010). Other approaches such as cross-over analysis, autocorrelation (Levine et al., 2002), and curve-fitting (Straume, 2004) could also be used for detection and evaluation of periodicity.

## Circadian Rhythm-Related Databases

Previous studies have reported that various tissues and cells exhibit CR *in vivo* or *in vitro*. As a growing number of tissues and cells have been found to display periodic gene expression, and the number of identified CCGs has increased. Based on accumulation of new data, researchers urgently need platforms to collect and obtain this information. Several databases have been constructed, including CircadiOmics^1^, CircaDB^2^, Bioclock^3^, SCNseq^4^, CGDB^5^, and CirGRDB^6^, which have different characteristics and functions. These databases allow investigators to search for genes of interest that exhibited rhythmic expression patterns in different species.

CircadiOmics contains circadian–related data sets from the livers of wild-type and Clock mutant mice, and is integrated with genomic, transcriptomic, proteomic, and metabolomic data sets (Patel et al., 2012). The CircaDB database contains circadian transcriptional profiles of over 3000 potential circadian genes in mice and humans (Pizarro et al., 2013). Bioclock is a s resource library which contains diel and circadian microarray expression data from *Aedes aegypti* (1674 potential circadian genes) and *Anopheles gambiae* (∼1000 potential circadian genes) (Leming et al., 2014). SCNseq is an important chronobiological resource that contains data from mouse SCN, and contains 4569 rhythmic genes and 3187 intergenic non-coding RNAs (Pembroke et al., 2015).

Among the above databases, Bioclock is focused on insects (mosquitoes), and CircadiOmics, SCNseq, and CircaDB are focused on mammals (mouse and human). However, these databases are still not considered comprehensive despite having been established for several years. CGDB, a new database of cycling genes in eukaryotes established in recent years, contains ∼73,000 oscillating genes across phyla (68 animals, 39 plants, and 41 fungi) from published small-scale or high-throughput data obtained from various species (Li et al., 2017). This database can be browsed “by species” or “by external conditions,” and genes can be reported in peaks or troughs at specific time points (Li et al., 2017). In addition, another new database, CirGRDB, has integrated over than 4936 genome-wide assays, and allows users to retrieve three groups of transcriptome profiles including normal conditions (oscillatory patterns in normal tissues or cell lines), different conditions (oscillatory patterns under different conditions), and other conditions (expression pattern of specific genes under knock-out/down or over-expression) (Li et al., 2018). This database also provides information about regulatory mechanisms including transcriptional factors, histone modification, chromatin accessibility, enhancer RNAs, microRNAs, RNA binding proteins, RNA editing, and RNA methylation, and can be used to construct a regulatory network of rhythmic genes at multiple regulatory layers (Li et al., 2018). The CirGRDB database adds information about post-translational modifications, transcriptional factors, and epigenetic modifications that were not available in previous databases. These databases that integrate circadian information have been established successively in recent years, and each have different characteristics and functions. These databases allow for convenient evaluation of past results, which may provide scientific direction for future studies.

## Experimental Causes of Circadian Rhythm Disorders

After synchronizing the CR by appropriate means and monitoring the circadian oscillations of subjects, any inconsistency in our observations compared with previous results requires careful evaluation of the experimental conditions, since there are lots of factors that can influence the circadian clock.

Light, food, temperature, arousal stimulation, and oxygen concentration in the environment can affect CR in mammals. As previously described, exposure light pulses, different photoperiods, constant light conditions, differing light intensity, and different wavelengths of light can alter the phase, period, or amplitude of CR. In addition to entrainment, negative masking is another response of animals to light. Thus, in CR experiments researchers must control all appropriate parameters and avoid negative masking.

As the previous description, time-restricted feeding entrains the clock, and shifts the phase of peripheral clocks from that observed with *ad libitum* feeding (Damiola et al., 2000; Stokkan et al., 2001) and calorie restriction (Mendoza et al., 2008; Patel et al., 2016). In addition, different ingredients in food can cause inconsistencies in circadian rhythm expression patterns. For example, high fat diet impacts the central and peripheral clocks, and significantly decreases the amplitude of circadian oscillations in the liver (Kohsaka et al., 2007). This may be due to elevated blood glucose levels, insulin resistance, or other metabolic changes (Bass and Takahashi, 2010). Moreover, high fat diet signals could promote release of gastrointestinal tract-derived peptides or bile, resulting in dysregulation of PPAR-α signaling in the liver and other tissues, which may explain high fat diet-induced rhythm disruption (Asher et al., 2010; Zarrinpar et al., 2016). Timed restricted feeding resets the circadian oscillations of animals on a high fat diet and prevents high-fat diet-caused obesity through improving CREB, mTOR, and AMPK pathway function (Hatori et al., 2012; Sherman et al., 2012). High salt diet also influences clock oscillations. Two weeks of 4% NaCl administration resulted in phase advances in mouse peripheral clocks (Oike et al., 2010). Therefore, it is necessary to control the ingredients and caloric value of the diet, and the feeding approach (time-restricted feeding or *ad libitum* feeding), to control the peripheral oscillators. Although other non-photic entrainment factors such as arousal stimuli, ambient temperature and oxygen concentration have weaker effects on the animal CR system (previously described), it is still necessary to control all the above factors to maintain consistent and reproducible experimental conditions.

Causes of circadian rhythm disturbance in cultured cells or tissues include cell density, osmotic pressure, media PH, mechano-environment, temperature, oxygen concentration, and microorganisms. The circadian amplitude depends on cell density. The rhythmicity of low-density SCN neurons and fibroblasts was greatly reduced, and was significantly enhanced in high-density cultures (Liu et al., 2007). Coupling between SCN neurons could enhance the rhythmicity of a cell population (Liu et al., 2007), and the normal rhythmic expression of fibroblasts need paracrine signals from adjacent cells (Noguchi et al., 2013). Moreover, a stiff extracellular matrix increases the amplitudes of circadian oscillations in mammary epithelial cells, and decreases in amplitudes in mammary fibroblasts occur through mechanotransduction pathways mediated by integrin adhesion and Rho signaling (Yang et al., 2017; Williams et al., 2018). Furthermore, the circadian period can be regulated by osmotic stress. A previous report showed that the circadian period of mouse embryonic fibroblasts can be lengthened using hypertonic media and shortened using hypotonic media through ASK -dependent phosphorylation of proteins (Imamura et al., 2018). Furthermore, an intense alter in media pH (±0.4) induces extensive phase shifts (>8 h) of clock genes in rat fibroblasts, likely through the TGF-β signaling pathway (Kon et al., 2008). As previously described, circadian oscillations can be entrained by changes in ambient temperature. In addition, temperature pulses for 1 or 6 h also cause phase shifts in *ex vivo* pituitary or lung cultures (Kon et al., 2008). Furthermore, oxygen levels affect CR. These results imply that it is crucial to control cell density, the mechano-environment, osmotic pressure, pH, and oxygen concentration when evaluating circadian rhythms. Cells contaminated microorganisms can exhibit circadian genes expression disruption. The presence of bacteria can reduce the amplitudes circadian rhythms in mouse enterocytes (Mukherji et al., 2013; Wang et al., 2017) and lung epithelial cells (Haspel et al., 2014) by directly invading or secreting endotoxins that activate the TLR signaling pathway. Moreover, multiple viruses such as Hepatitis B and C viruses, human immunodeficiency virus, Coxsackie virus A16, human T-lymphocyte virus type 1, and influenza can also disrupt the expression of clock genes (Zhuang et al., 2017). Therefore, it is necessary to prevent microbial contamination when evaluating CR.

In addition to the above environmental factors, the age of animals or cells should also be considered when studying CR. Circadian rhythm oscillations in humans and rodents gradually dampen with increasing age, although whether this age-related change is owing to dysfunction of inherent core clock or insufficient environmental entrainment is unclear (Chang and Guarente, 2013; Wang et al., 2015). In general, CR can be affected by many environmental factors. Any imbalance of these factors can lead to circadian disorders, which can contribute to several diseases through a series of signal pathways and molecular mechanisms.

## Circadian Rhythm Disorder and Diseases

In mammals, many physiological functions under the regulation of the circadian clock are affected by external cues such as sleep and wakefulness, alertness and motor ability, body temperature fluctuations, the urinary system, hormone secretion, immune regulation, cytokine release, and cell cycle progression (Panda et al., 2002; Bass and Takahashi, 2010). When circadian systems are disrupted by various environmental or genetic defects, dysfunction of various physiological processes can occur (Hastings et al., 2003).

A common reason of human circadian disorder is misalignment between the environmental rhythm (e.g., light–night cycle) and the endogenous circadian oscillators. Previous reports have shown that human subjects who were maintained in a controlled circadian misalignment condition experienced severe imbalances in glucose homeostasis, insulin action, and appetite control (Buxton et al., 2012; McHill et al., 2014). In animal studies, when the intrinsic clocks of animals are desynchronized from external timing cues, disorders in many organs or tissues can occur, including diet-induced obesity (Kohsaka et al., 2007; Arble et al., 2009), a light-induced pro-inflammatory state (Lucassen et al., 2016), cardiac fibrosis, and systolic dysfunction (Penev et al., 1998; Martino et al., 2008). Genetically, since the molecular clock is highly conserved, diseases caused by mutations of clock genes (which are often used in animal models to study clock gene-related dysfunction) are rare in humans. An examples of these kinds of mutations is familial advanced sleep phase syndrome, which is induced by a missense mutation (S662G) of the core clock gene *PER2* (Jones et al., 1999; Reid et al., 2001). In addition, several clock gene single nucleotide polymorphisms related to metabolic syndrome, hypertension, and diabetes mellitus also have been identified in genome-wide association studies (Saxena et al., 2007; Woon et al., 2007; Zeggini et al., 2007; Scott et al., 2008).

A large quantity of studies have suggested that circadian disruptions are closely associated with the formation and development of various diseases including cancer (Momma et al., 2017; Polo et al., 2017; Xiong et al., 2018), dysplasia (Vilches et al., 2014; Voiculescu et al., 2016; Zhao et al., 2018; Zhou et al., 2018), cardiovascular disease (Angelousi et al., 2018), obesity (Antunes et al., 2010), diabetes (Pan et al., 2011), and sleep disorder (Logan and McClung, 2019). As previously described, the cell cycle and the circadian clock are two basic periodic processes of a day, and they are interrelated. Clock genes contribute to the occurrence and development of tumors by regulating and interfering with cell cycle-related genes such as *c-Myc*, *P53*, and *P21* (Angelousi et al., 2018). In addition, our previous studies also suggested that circadian disruption led to an abnormal increase in the precancerous gene *PFKFB3*, thereby promoting differentiation from normal cells to tumor cells (Chen et al., 2016). Further, deletion of the core clock protein BMAL1 attenuated inhibition of TERT transcription, and TERT upregulation is closely related to tumorigenesis (Tang et al., 2017).

Disorders of the circadian clock also affect the development of organs such as the brain and bone, leading to dysplasia. CR are essential for brain development (Novakova et al., 2010). Studies have indicated that rhythms of clock genes and genes encoding NMDA receptor subunits are inhibited in the hippocampus of rat adult progeny subjected to persistent illumination during pregnancy, resulting in impaired spatial memory in these animals (Vilches et al., 2014; Voiculescu et al., 2016). Further, recent human and animal studies suggested that sleep and circadian disruptions during pubescence can impact brain development and may lead to susceptibility to mood and substance use disorders (Logan and McClung, 2019). Moreover, circadian clocks participate in regulation of neurogenesis. Studies have displayed that the clock component REV-ERBα directly inhibited the promoter of the *FABP7* gene, a marker for neuronal progenitor cells, resulting in alterations in neuronal differentiation (Young et al., 2013; Giachino et al., 2014). Loss of REV-ERBα resulted in increased *FABP7* expression and hippocampal neurogenesis, which was in connection with changes in mood-related behaviors (Schnell et al., 2014). Furthermore, our previous studies showed the importance of the circadian clock on bone development. Circadian disruption resulted in *Bmal1* down-regulation, leading to direct inhibition of *Opg* transcription (Zhou et al., 2018) and an indirect increase in *Mmp3* expression through P65 phosphorylation (Zhao et al., 2018). These changes promoted osteoclasis and suppressed osteogenesis, resulting in bone loss and abnormal mandibular development.

Circadian-related metabolic diseases may be associated with defective glucose tolerance and insulin resistance, and abnormal glucose metabolism (Stenvers et al., 2018). Circadian disruption may result in aberrant glucocorticoid and melatonin levels, which could affect insulin secretion (glucocorticoids decrease insulin secretion, and melatonin increases insulin secretion) (Stenvers et al., 2018). In addition, a study exhibited that the circadian clock may maintain long time energy balance through regulating the leptin endocrine feedback loop between adipose tissue and the brain, as demonstrated by circadian clock defects or long-time jet lag resulting in leptin-resistant animals (Kettner et al., 2015). Moreover, other studies indicated that the circadian clock regulates levels of neurohormones involved in cardiovascular function such as angiotensin II, renin, aldosterone, growth hormone, and atrial natriuretic peptide (Bhatnagar, 2017). These neurohormones may be responsible for increased blood pressure and inflammation in response to circadian misalignment (Scheer et al., 2009; Morris et al., 2015).

Higher incidences of brain diseases such as sleep disorders and depression were observed in a population with interrupted CR (Logan and McClung, 2019). Moreover, circadian dysregulation can induce emotional and psychotic attacks in individuals already suffering from psychiatric disorders (Logan and McClung, 2019). Several processes such as oxidative stress, inflammation, dopamine synthesis, and cellular metabolism are under the control of the circadian clock, and may contribute to neurodegeneration (Logan and McClung, 2019). Although most studies have suggested that circadian disorder is a symptom of neurodegenerative diseases including Alzheimer’s disease and Parkinson’s disease, some evidence indicates that circadian dysregulation induced by night illumination can cause enhanced incidence of tau deposition and neurodegeneration (Kim et al., 2018). Furthermore, increased risk of Alzheimer’s disease and Parkinson’s disease has been associated with the incidence of single-nucleotide polymorphisms in *CLOCK* and *BMAL1*, and in *BMAL1* and *PER1*, respectively (Logan and McClung, 2019). Therefore, circadian rhythm disorders may act on accelerating the progression of diseases in vulnerable individuals.

Taken together, many studies have highlighted that increased risk of multiple diseases including carcinoma, dysplasia, metabolic disorders, and neurodegenerative diseases are associated with circadian disorders induced by changes in external environmental cues. The mechanisms responsible for these associations may be physiological processes including cell proliferation, differentiation, oxidative stress, inflammation, synthesis, and cellular metabolism, which are regulated by the circadian clock.

## Conclusion and Future Prospects

Circadian clock system plays vital roles in the regulation of physiological processes, including cell cycle progression, cytokine release, hormone secretion, sleep and wakefulness, immune regulation, etc. Multiple systemic diseases are proven to be closely associated with circadian rhythm disorders, so chronobiology research is becoming a focal point in biological and medical fields at present. With the development of chronobiology in these years, external cues that can be used as synchronizers to reset the circadian oscillators of animals or cells have been gradually discovered. From time-serial collection to real-time monitoring through luciferase reporter genes and fluorescent proteins, the methods to observe biological rhythms are rapidly advancing and becoming more diverse. In addition, researchers have also established a number of circadian clock-related databases to facilitate access to previous research results. Through the combination of various *in vivo* and *in vitro* experiments, the mechanisms underlying circadian oscillations are constantly being elucidated, and the complicated connections between circadian rhythm disorders and various diseases are also being identified. Illuminating the crosstalk between circadian rhythm and human diseases can help us better clarify the pathogenesis of circadian-related diseases, which provides new strategies and ideas for disease prevention and treatment.

## Author Contributions

YX and QT contributed equally to this review. LC, QT, and YX conceived and wrote the manuscript. GC, MX, and SY contributed to data acquisition, analysis, and interpretation. LC and JZ critically revised the manuscript.

## Conflict of Interest Statement

The authors declare that the research was conducted in the absence of any commercial or financial relationships that could be construed as a potential conflict of interest.

## Footnotes

1 http://circadiomics.igb.uci.edu/

2 http://circadb.hogeneschlab.org/

3 https://www3.nd.edu/~bioclock/

4 www.wgpembroke.com/shiny/SCNseq/

5 http://cgdb.biocuckoo.org/

6 http://cirgrdb.biols.ac.cn/

## Abbreviations

βOHB: beta-hydroxybutyrate
AMPA: α-amino -3-hydroxy-5-methyl-4-isoxazolepropionic acid
AMPK: adenosine monophosphate-activated protein kinase
ASK: apoptosis signal-regulating kinase
AVP: arginine vasopressin
BMAL1: brain and muscle Arnt-like protein 1
CaMK: calcium-calmodulin kinase
cAMP: cyclic adenosine monophosphate
CCD: charge-coupled device
CCGs: clock-controlled genes
CIRBPs: cold-inducible RNA binding proteins
CLOCK: circadian locomotor output cycles kaput
CR: circadian rhythms
CRE: cAMP response elements
CREB: cAMP response element-binding protein
Cry: cryptochrome
CT: circadian time
DBP: D-box binding protein
DD: dark-dark
Dex: dexamethasone
DM: dorsomedial
DPSC: dental pulp-derived stem cells
E4BP4: E4 Promoter-Binding Protein 4
EGF: epidermal growth factor
EGR1: Early growth response protein 1
FAA: food anticipatory activity
FABP7: fatty acid binding protein 7
FEO: food-entrainable oscillator
FFT: fast fourier transform
Fsk: forskolin
GABA: gamma-aminobutyric acid
GHT: geniculohypothalamic tract
GR: glucocorticoid receptor
GRE: glucocorticoid response element
HLF: hepatic leukemia factor
HSF1: heat shock factor 1
IGL: intergeniculate leaflet
IRC: irradiance response curve
LD: light-dark
MAPK pathway: mitogen-activated protein kinase pathway
Mmp3: matrix metalloproteinase 3
MRTFs: myocardin-related transcription factors
mTOR: mammalian target of rapamycin
NADH: nicotinamide adenine dinucleotide
NADPH: nicotinamide adenine dinucleotide phosphate
NFIL3: nuclear factor interleukin-3-regulated protein
NMDA: N-methyl D-aspartate
NPAS2: neuronal PAS domain protein 2
NPY: neuropeptide Y
Opg: osteoprotegerin
PAC1: proteasome assembly chaperone 1
PACAP: pituitary adenylyl cyclase activating peptide
PAR-bZip: proline and acidic amino acid-rich basic leucine zipper
PCR: polymerase chain reaction
Per: Period
PGE2: prostaglandin E2
PKA: protein kinase A
PKCA: protein kinase Cα
PKG: protein kinase G
PMTs: photomultiplier tubes
PPAR: peroxisome proliferator-activated receptors
pRGC: photosensitive retinal ganglion cell
RHT: retinohypothalamic tract
ROREs: ROR binding elements
RORs: retinoic acid receptor-related orphan receptors
SCN: suprachiasmatic nucleus
SP: substance P
TEF: thyrotroph embryonic factor
TERT: telomerase reverse transcriptase
TGF-β: transforming growth factor beta
TLR: toll-like receptor
TRP channel: transient-receptor potential channel
TRPCs: transient-receptor potential channels
TTFLs: transcription and translation feedback loops
VIP: vasoactive intestinal polypeptide
VL: ventrolateral
VPAC2: vasoactive intestinal peptide receptor 2
ZT: zeitgeber time.
